# The pathogens profile in children with otitis media with effusion and adenoid hypertrophy

**DOI:** 10.1371/journal.pone.0171049

**Published:** 2017-02-23

**Authors:** G. P. Buzatto, E. Tamashiro, J. L. Proenca-Modena, T. H. Saturno, M. C. Prates, T. B. Gagliardi, L. R. Carenzi, E. T. Massuda, M. A. Hyppolito, F. C. P. Valera, E. Arruda, W. T. Anselmo-Lima

**Affiliations:** 1 Department of Ophthalmology, Otorhinolaryngology, and Head and Neck Surgery, Ribeirão Preto School of Medicine, University of São Paulo (USP), Ribeirão Preto, São Paulo, Brazil; 2 Department of Genetics, Evolution, and Bioagents, Institute of Biology, University of Campinas (UNICAMP), Biology Institute, Campinas, São Paulo, Brazil; 3 Department of Molecular and Cell Biology, Ribeirão Preto School of Medicine, University of São Paulo (USP), Ribeirão Preto, São Paulo, Brazil; University of Toledo College of Medicine and Life Sciences, UNITED STATES

## Abstract

**Objectives:**

To evaluate the presence of viruses and bacteria in middle ear and adenoids of patients with and without otitis media with effusion (OME).

**Methods:**

Adenoid samples and middle ear washes (MEW) were obtained from children with OME associated with adenoid hypertrophy undergoing adenoidectomy and tympanostomy, and compared to those obtained from patients undergoing cochlear implant surgery, as a control group. Specific DNA or RNA of 9 respiratory viruses (rhinovirus, influenza virus, picornavirus, syncytial respiratory virus, metapneumovirus, coronavirus, enterovirus, adenovirus and bocavirus) and 5 bacteria (*S*. *pneumoniae*, *H*. *influenzae*, *M*. *catarrhalis*, *P*. *aeruginosa* and *S*. *aureus*) were extracted and quantified by real-time PCR.

**Results:**

37 OME and 14 cochlear implant children were included in the study. At the adenoid, virus and bacteria were similarly detected in both OME and control patients. At the middle ear washes, however, a higher prevalence of bacteria was observed in patients with OME (p = 0.01). *S*. *pneumoniae* (p = 0.01) and *M*. *catarrhalis* (p = 0.022) were the bacteria responsible for this difference. Although total virus detection was not statistically different from controls at the middle ear washes (p = 0.065), adenovirus was detected in higher proportions in adenoid samples of OME patients than controls (p = 0.019).

**Conclusions:**

Despite both OME and control patients presented similar rates of viruses and bacteria at the adenoid, children with OME presented higher prevalence of *S*. *pneumonia*, *M*. *catarrhalis* in middle ear and adenovirus in adenoids when compared to controls. These findings could suggest that these pathogens could contribute to the fluid persistence in the middle ear.

## Introduction

Otitis media with effusion (OME) is a common childhood disease characterized by the presence of fluid in the middle ear, with no symptoms and/or signs of acute inflammation [1, 2]. In the United States, approximately 90% of all children develop an episode of OME before they reach school age, mainly between the ages of 4 months and 6 years [2]. The presence of OME is associated with severe negative impact on child development, including hearing loss with long-term consequences for speech and language acquisition, poor school performance, and imbalance issues [1–3]. Moreover, children with OME are five times more susceptible to develop acute otitis media than controls [4, 5].

The pathogenesis of OME is still not fully understood. Epidemiological data suggest that the pathogenesis of OME is multifactorial, involving anatomical, immunological, genetic, microbial, and environmental factors [6–9]. However, it is widely accepted that the dysfunction of the Eustachian tube play a key role in the development of OME in all ages.

Mechanical obstruction of the Eustachian tube is one of the main identifiable causes of Eustachian tube dysfunction, especially due to adenoid hypertrophy (AH) in the pediatric age. The presence of AH can obstruct the nasopharyngeal ostium of the Eustachian tube, leading to negative pressure in the middle ear cavity, and eventually mucosal transudation [10]. Indeed, AH is a frequent condition observed in patients with OME [7–9, 11]. In children with AH and OME, the surgical removal of the adenoid (associated with ventilation tube insertion) accelerates the recovery of the middle ear mucosa, decrease the risk of recurrence and need of repetitive surgical procedures, and reduce treatment failure rate. However, new evidence point out that the benefits of adenoidectomy in children with OME older than 4 years-old is independently of the adenoid size [12], suggesting that the removal of a local reservoir of pathogenic microbiota (viruses, planktonic bacteria, and biofilms) is the key for such benefits [13–15]. Despite some studies have evaluated the microbial colonization of adenoid and middle ear, no studies have thoroughly studied the association of respiratory pathogenic microbiota between these two niches. Therefore, the present study was carried out to compare the detection of common respiratory viruses and bacteria in adenoids and middle ear fluid in children with OME and in controls.

Moreover, adenoid can be reservoirs of pathogens that may reach the middle ear [13]. Adenoid samples obtained from patients with chronic adenoiditis are highly colonized by multiple species of viruses and bacteria. The rate of detection in hypertrophic adenoids is higher than 85% for viruses [14] and almost 100% for bacteria [15].

Bacteria and viruses have been detected in the middle ear fluid (MEF) from children with OME. Using an RT-PCR-based assay only for three respiratory viruses (rhinovirus, respiratory syncytial virus and human coronavirus), positivity was detected in 30% of MEF from children with OME in Finland [16]. In a study conducted in Pittsburgh, 75 of 97 (77.3%) samples of MEF from children with OME were positive by PCR for one or more of three bacteria (*M catarrhalis*, *H influenza* and *S pneumoniae*) [17].

## Methods

### Ethics statement

The study was conducted following the principles expressed in the Declaration of Helsinki, after approval by the Ethics Review Committee of the Clinical Hospital of the University of Sao Paulo School of Medicine in Ribeirão Preto, Brazil (#10466/2008). Written informed consent was obtained from all parents/guardians.

### Patients

The study enrolled patients seen at the Division of Otorhinolaryngology from May 2010 to August 2012. Two groups of patients were studied: a) children with OME and adenoid hypertrophy (AH) who underwent adenoidectomy and tympanostomy with ventilation tube insertion, and b) children with no middle ear disease, who underwent cochlear implantation due to severe hearing loss. All patients were evaluated by otoscopy, audiometry, tympanometry, and nasal endoscopy. OME was diagnosed by medical history suggesting persistence of middle ear fluid for more than 3 months without acute signs of inflammation, confirmed by the presence of tympanic membrane opacity, retraction, or air-liquid interface, and by a type B tympanogram and/or conductive hearing loss. AH was defined at nasal endoscopy by the presence of adenoid in contact with the torus tubaris (grade 3) and/or vomer (grade 4) [18, 19]. Exclusion criteria were: prior surgical procedures in the upper airways, including tympanostomy for tube placement; use of antibiotics or symptoms of respiratory infection within the last month; history of recurrent acute otitis media or recurrent upper airways infection; tympanic membrane perforation, or the presence of genetic syndromes, such as Down syndrome. All patients with OME and AH were under treatment with nasal corticosteroids for at least one month during the preoperative period.

The control group consisted of children undergoing cochlear implant due to severe hearing loss with no medical history of otitis media, no respiratory infection within the last month, absence of tympanometric alterations, nor adenotonsillar hypertrophy.

All patients followed the vaccination protocols of the Brazilian ministry of health [20], which includes some pathogens targeted in the present study, such as pneumococcal conjugated. Additionally, patients in the control group were immunized with pneumococcus 23-valent vaccine and meningococcus C, according to the cochlear implant surgery protocol.

### Specimen collection

Fragments of adenoid (surface and stroma) and middle ear washes (MEW) were obtained from all patients under general anesthesia. Briefly, adenoids fragments were collected using Beckmann adenoid curette (from OME-AH patients) or punch biopsy forceps, under direct visualization after soft palate retraction, in order to avoid contamination (controls). Adenoids samples were placed in Eagle’s minimal essential medium (MEM) with 15% of a solution containing 20,000 U/mL of penicillin-streptomycin and 200 μg/mL of amphotericin B, 10% of fetal bovine serum (Gibco^®^, Life Technologies, Carlsbad, CA, USA), and kept on ice until further processing. Adenoids were washed twice in MEM to remove blood and tissue debris and the fragment was macerated in Trizol^®^ (Invitrogen, Life Technologies, Carlsbad, CA, USA) for later nucleic acid extraction.

In children with OME, MEW was performed with 0.5 mL of sterile saline through a small tympanostomy using a sterile needle coupled to a 5 mL syringe, after mechanical cleaning of the external ear canal. After that, the MEW was conditioned into a polypropylene microtube and treated with antimycotic and antibiotic solution (Gibco^®^, Grand Island, NY, USA) for one hour at 4°C. Aliquots were made in thrice the volume of Trizol, and frozen at −80°C until further processing. From each patient, samples were collected from only one side, which was randomly chosen when bilateral disease was present.

In control patients, MEW was obtained after partial mastoidectomy was performed, when direct access to the middle ear was possible through the attic, with the same sterile technique utilized to collect samples in OME patients.

### Nucleic acid extraction and pathogen detection

RNA was extracted from 250 μL of MEW or from approximately 30 mg of adenoid tissue using 750 μL of Trizol^®^ [21], according to the manufacturer’s protocol. DNA was further extracted using DNA purification kit (Promega^®^, Fitchburg, WI, USA) starting with the DNA-enriched fraction obtained with Trizol^®^. Samples were tested by real-time PCR for rhinovirus (HRV), influenza virus (FLU), parainfluenza virus (HPIV), syncytial respiratory virus (HRSV), metapneumovirus (HMPV), coronavirus (HCoV), enterovirus (HEV), adenovirus (HAdV) and human bocavirus (HBoV) following an in house real-time PCR protocol, based on primers listed on S1 Table and TaqMan probes, with the same procedures published elsewhere (14]. Real-time PCR was also used to detect the presence of *S*. *pneumoniae*, *H*. *influenzae*, *M*. *catarrhalis*, *P*. *aeruginosa*, and *S*. *aureus*, using the primers listed on S2 Table.

## Statistical analysis

The Fisher’s exact test was used to compare rates of virus and bacteria detection in both groups of patients. Wilcoxon test was done to assess correlation between bacteria and virus detected in each patient. The analysis was performed using GraphPad Prism 5 (La Jolla, CA, USA).

## Results

### Clinical data

14 control patients (2–12 years-old; mean 4 years 6 months; SD 2.64; 9 male / 5 female) and 37 children with OME and AH (2–12 years-old; mean 6 years 1 month; SD 1,97; 19 male / 18 female) were included in this study.

No severe complications related to the sampling procedure, such as nasal or oral bleeding, dysphagia and otorrhea, were observed in the control group as well as in the OME-AH group within the first 48 hours after surgery or after 3 weeks postoperative reassessment.

### Detection of viruses

At least one virus species was detected, considering adenoid samples, in 32 of 37 (86.5%) patients with OME-AH and in 11 of 14 (78.6%) control patients. Differently, at least one virus was detected in MEWs from 19 of 37 (51.3%) patients with OME, and in 3 of 14 (21.4%) control patients.

The overall rates of virus detection were significantly higher in adenoids as compared to MEW in both groups (p = 0.002 for the OME group and p = 0.007 for the control group), but the virus detection rates in MEW and adenoids were not significantly different between patients with OME and control children (respectively p = 0.06 and p = 0.66). Despite this fact, HAdV was more often found in adenoids of OME-AH group as compared to controls (p = 0.01) (Table 1).

**Table 1:** Viral detection in adenoid and middle ear samples from OME and control patients.

	OME Patients		Control Patients		p-value	
Virus	Adenoid	Middle ear	Adenoid	Middle ear	Adenoid	Middle Ear
	N (%)	N (%)	N (%)	N (%)		
**HEV**	17 (45.9)	13 (35.1)	7 (50.0)	2 (14.2)	P = 1.00	P = 0.18
**HRV**	5 (13.5)	2 (5.4)	2 (14.2)	1 (7.1)	P = 1.00	P = 1.00
**HRSV**	3 (8.1)	0 (0)	4 (28.5)	0 (0)	P = 0.07	P = 1.00
**HMPV**	3 (8.1)	5 (13.5)	2 (14.2)	0 (0)	P = 0.60	P = 0.30
**HPIV**	0 (0)	0 (0)	2 (14.2)	0 (0)	P = 0.07	P = 1.00
**FLU**	0 (0)	0 (0)	0 (0.0)	0 (0)	P = 1.00	P = 1.00
**HCoV**	4 (10.8)	0 (0)	0 (0.0)	0 (0)	P = 0.56	P = 1.00
**HBoV**	6 (16.2)	1 (2.7)	1 (7.1)	0 (0)	P = 0.65	P = 1.00
**HAdV**	16 (43.2)	0 (0)	1 (7.1)	0 (0)	P = 0.01*	P = 1.00
**Total**	32 (86.5)	19 (51.3)	11 (78.6)	3 (21.4)	P = 0.66	P = 0.06

*Valid statistical significance

**Fig 1:**
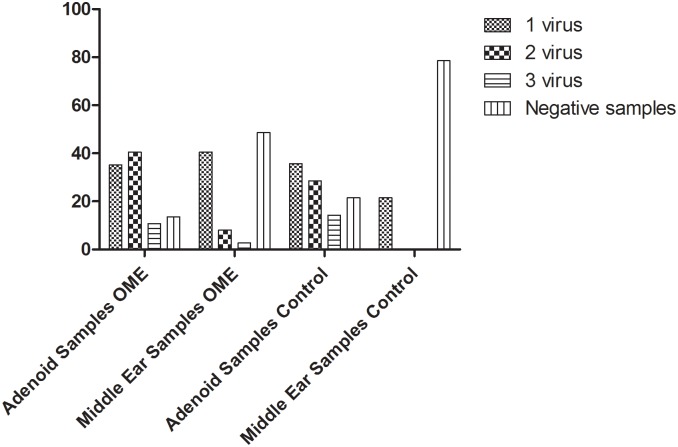
Percentages of single infections and co-infections by respiratory viruses in adenoids and middle ear samples from OME and control patients.

The rates of virus co-detection in positive samples were 59.3% (19/32) in adenoids and 21% (4/19) in MEWs. Two or 3 viruses were simultaneously detected in adenoid tissues from 15 (40.5%) and 4 (10.8%) patients with OME. In contrast, only 3 (8.1%) control patients had 2 viruses and only 1 (2.7%) had 3 different viruses detected in MEWs (Fig 1). There was no significant concordance between detection of viruses in adenoid and the middle ear (p = 0.65). Only 6 (16.2%) patients with OME (5 HEV and 1 HBoV) and 1 control patient (HEV) had the same virus detected in MEWs and adenoid tissue, suggesting that the viral colonization of the middle ear is independent from viral infection of adenoids.

### Detection of bacteria

Notably, the rates of bacteria detection in MEWs was significantly higher in patients with OME than in control patients (p = 0.01), especially due to *S*. *pneumococcus* and *M*. *catarrhalis*. Overall bacterial detection rates in adenoids were similar between OME and control patients (p = 0.29). Twelve of 14 control patients (85.7%) had at least one species of respiratory pathogenic bacteria detected in adenoid tissues, reinforcing previous data that indicate that potentially pathogenic bacteria colonize adenoids of healthy children [22, 23] (Table 2).

**Table 2:** Bacterial detection in adenoid and middle ear samples from OME and control patients.

Bacteria	OME Patients		Control Patients		Significance	
	Adenoid	Middle ear	Adenoid	Middle ear	AD	ME
	N (%)	N (%)	N (%)	N (%)	P value	P value
***S*. *pneumoniae***	21 (56.7)	13 (35.1)	9 (64.2)	0 (0)	0.75	0.01*
***H*. *influenzae***	16 (43.2)	7 (18.9)	9 (64.2)	2 (14.2)	0.22	1.00
***M*. *catarrhalis***	15 (40.5)	12 (32.4)	4 (28.5)	0 (0)	0.52	0.02*
***P*. *aeruginosa***	8 (21.6)	11 (29.7)	2 (14.2)	1 (7.1)	0.7	0.14
***S*. *aureus***	1 (2.7)	1 (2.7)	2 (14.2)	0 (0)	0.17	1.00
**TOTAL**	25 (67.5)	24 (64.8)	12 (85.7)	3 (21.4)	0.29	0.001*

*Valid statistical significance

**Fig 2:**
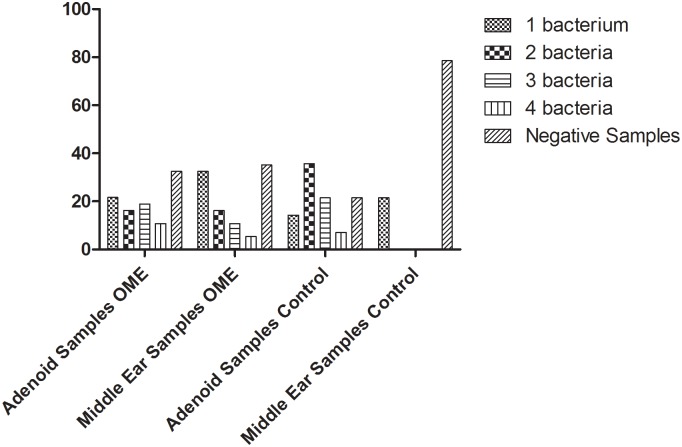
Percentages of single infections and co-infections by potentially pathogenic bacteria in adenoids and middle ear samples from OME and control patients.

With regard to bacteria co-detection in adenoids and MEWs from OME patients, 18 patients (48.6%) with OME had more than one bacterium detected in adenoid, and two, three, or four bacteria were simultaneously detected respectively in 16.2%, 18.9%, and 13.5% of patients. Six patients had 2 (16.2%), four had 3 (10.8%) and two patients had 4 bacteria (5.4%) simultaneously detected in MEWs for an overall frequency co-detection of bacteria in MEWs of 32.4% (12 of 37 patients). Bacteria co-detection was also frequent in the control group, in which five patients had 2, three patients had 3 and one patient had 4 different bacteria simultaneously detected in adenoid tissues. Bacteria co-detection was not observed in MEWs from control patients (Fig 2).

Similar to the findings regarding viruses, there was no concordance between bacteria detected in adenoids and MEWs, both in controls and OME patients (p = 0.18). The same bacteria were detected in the MEWs and adenoids was observed in 13 (35.1%) patients with OME, and in 2 (14.2%) controls (Fig 2).

### Association between virus and bacteria detection

Multiple associations between viruses and bacteria in the adenoid and middle ear were investigated. The only microbes that presented positive association was *S*. *pneumoniae* and HAdV in hypertrophic adenoids of patients with OME (p = 0.02) (Tables 3 and 4). We did not find any significant association between virus and bacteria in the adenoid or middle ear in patients of the control group.

**Table 3:** Detection of respiratory viruses and bacteria in adenoid samples in patients with OME.

Adenoid—OME	*S*. *Pneumoniae*	*H*. *influenzae*	*M*. *catarrhalis*	*P*. *aeruginosa*	*S*. *aureus*	Total
	N (%)	N (%)	N (%)	N (%)	N (%)	N (%)
HAdV	11 (29.7)*	7 (18.9)	7 (18.9)	4 (10.8)	0 (0)	13 (35.1)
HBoV	3 (8.1)	4 (10.8)	3 (8.1)	4 (10.8)	0 (0)	6 (16.2)
HRV	4 (10.8)	3 (8.1)	3 (8.1)	1 (2.7)	0 (0)	4 (10.8)
HEV	10 (27.0)	6 (16.2)	5 (13.5)	2 (5.4)	1 (2.7)	11 (29.7)
HRSV	2 (5.4)	2 (5.4)	2 (5.4)	0 (0)	0 (0)	2 (5.4)
HMPV	2 (5.4)	2 (5.4)	1 (2.7)	0 (0)	0 (0)	2 (5.4)
FLU	0 (0)	0 (0)	0 (0)	0 (0)	0 (0)	0 (0)
HPIV	0 (0)	0 (0)	0 (0)	0 (0)	0 (0)	0 (0)
HCoV	2 (5.4)	2 (5.4)	1 (2.7)	0 (0)	0 (0)	2 (5.4)
Total	20 (54.0)	15 (40.5)	14 (37.8)	8 (21.6)	1 (2.7)	24 (64.8)

*Valid statistical significance

**Table 4:** Detection of respiratory viruses and bacteria in adenoid samples from control patients undergoing cochlear implantation.

Adenoid—Control	*S*. *Pneumoniae*	*H*. *influenzae*	*M*. *catarrhalis*	*P*. *aeruginosa*	*S*. *aureus*	Total
	N (%)	N (%)	N (%)	N (%)	N (%)	N (%)
HAdV	0 (0)*	1 (7.1)	1 (7.1)	0 (0)	0 (0)	1 (7.1)
HBoV	0 (0)	0 (0)	0 (0)	0 (0)	0 (0)	0 (0)
HRV	1 (7.1)	2 (14.2)	2 (14.2)	0 (0)	0 (0)	2 (14.2)
HEV	5 (35.7)	4 (28.5)	2 (14.2)	1 (7.1)	0	6 (42.8)
HRSV	2 (14.2)	2 (14.2)	1 (7.1)	1 (7.1)	0	3 (21.4)
HMPV	0 (0)	0 (0)	0 (0)	0 (0)	0 (0)	0 (0)
FLU	0 (0)	0 (0)	0 (0)	0 (0)	0 (0)	0 (0)
HPIV	2 (14.2)	1 (7.1)	1 (7.1)	2 (14.2)	0 (0)	2 (14.2)
HCoV	0 (0)	0 (0)	0 (0)	0 (0)	0 (0)	0 (0)
Total	7 (50.0)	6 (42.8)	3 (21.4)	2 (14.2)	0 (0)	9 (64.2)

*Valid statistical significance

## Discussion

Although the pathogenesis of OME is not fully understood, there is evidence that adenoid may play an important role in this disease, either by mechanically impairing the Eustachian tube function, or by acting as a microbial reservoir for ascending infection to the middle ear [13, 14, 24]. Children are often exposed to pathogens that may chronically persist in tissues in the upper airways, especially in the adenoid and tonsils [13, 14, 25, 26]. Thus, as the adenoid has an intimate anatomical relation with the Eustachian tube and ultimately to the middle ear, it is important to understand how the presence of microbes in the adenoid could lead to the development or persistence of OME in children.

To the best of our knowledge, this is the first case-control study that compared adenoid and middle ear microbiota of OME patients with healthy children. In our study, we used a sensitive method to detect nucleic acid of a comprehensive panel of respiratory viruses and bacteria to compare the microbial colonization of adenoid and its correspondence in the middle ear in both OME children and controls.

The overall detection rate of bacteria in the middle ear was significantly higher in patients with OME than in controls, but was similar for viruses. The higher overall frequency of bacteria detection in MEWs from OME patients was expected, since the accumulation of middle ear effusion favors growing of bacterial elements that may have been suctioned through the tube from the nasopharyngeal microbiota. Moreover, these bacteria may play roles as chronic inflammatory stimuli to the mucosa of the middle ear and contribute to the dysfunction of Eustachian tube. There was little or no concordance of the microbe detected in the adenoid and the middle ear, both in patients with OME as well as in controls. This finding is in agreement with a previous report that performed 16S rDNA pyrosequencing of both niches, showing that the adenoid microbiota was more diverse and complex than of the middle ear in a child with OME [27]. As the adenoid is exposed to the nasal and oral microbial contents, differently from the enclosed cavity of the middle ear, this makes the adenoid more susceptible to be colonized by a higher load and more variety of microorganisms. It is generally accepted that the development of negative pressure within the middle ear cavity suctions microbial components of the nasopharyngeal microbiota but, at present, there is no enough data to rule out the establishment of a selected microbiome especial to the middle ear cavity.

Interestingly, adenovirus detection was more frequent in hypertrophic adenoid tissues from patients with OME than in normal adenoids from healthy controls. This is in agreement with a paper previously published by our group reporting high rates of adenovirus detection by PCR in hypertrophic adenoids [14]. It has not been clear whether adenovirus has any role in pathogenesis of adenoid hypertrophy, especially because this virus is also frequent in nasopharyngeal secretions from healthy individuals [28]. Since some HAdV species are more prone than others to have more prolonged shedding [29], prospective longitudinal studies of viruses in patients with hypertrophic tonsils should include determination of adenovirus species to evaluate whether they are associated with chronic tonsillar hypertrophy.

The observed association of HAdV with *S*. *pneumoniae* in adenoids could lead to speculate that the epithelial damage induced by HAdV could somehow favor the secondary local proliferation of *S*. *pneumoniae* [30]. Some viruses, for instance influenza, are known to favor pneumococcal colonization of the upper airways by multiple mechanisms, including exposure of receptors for pneumococcal adherence and by providing nutrients sources for bacterial growth [31]. However, the simultaneous presence of HAdV and *S*. *pneumoniae* in adenoid tissue could be independent observations, due to the high frequencies of both microorganisms in the adenoid.

*S*. *pneumoniae* and *M*. *catarrhalis* were detected more often in middle ear washes from OME patients than controls. Considering that *S*. *pneumoniae* and *M*. *catarrhalis* are the most frequent pathogens detected in acute otitis media [6], and are able to ascend through the Eustachian tube [32], causing ciliary damage to the airway epithelium and disrupting mucociliary flow, this may result in conditions for persistence in the middle ear compartment [33–36]. In some cases, especially when OME is not clearly related to a mechanical obstruction of the Eustachian tube, the Toynbee effect of negative pressure within the middle ear may help ascendance of microbes through the tube, leading to colonization of the middle ear, which may be pivotal in OME pathogenesis.

*M*. *catarrhalis* has been widely studied because of its importance in middle ear diseases, and this interest has even led to vaccines for *M*. *catarrhalis* being proposed [37]. It’s mechanisms of adherence and triggering of the immune response have been well studied and even when not related to clinically relevant infection processes, appears with a high level of colonization during childhood [38]. Once regarded as nonpathogenic, *M*. *catarrhalis* could be related to several chronic affections of the upper airways [39].

Microorganisms in sessile biofilms are frequently resistant to the innate and adaptive immune responses and to antimicrobial agents. In OME, there has been evidence that the presence of middle ear fluid is associated to formation of biofilm in the middle ear, and that these biofilms are related to the local inflammation [9, 24]. In conventional, culture-based microbiology, detection of these microorganisms is not always possible, and the use of sensitive molecular methods, such as real-time PCR, enables detection of multiple microbes in complex niches like the middle ear. Although PCR cannot distinguish viable from dead microbes, the specificity of the primers coupled with careful sampling technique in order to minimize cross-contamination between sampling sites, ensure that the microorganisms were indeed present in the middle ear.

Viruses and bacteria interact in many different ways on different mucosal surfaces [40], including the upper respiratory tract mucosa and tonsils [41]. Persistence of viral infections includes a complete reprogramming of the local immune response, attenuation of production of type 1 interferon, and local alteration of the CD4+/CD8+ T cell balance [41, 42]. The airway mucosa affected by viral infection becomes more susceptible to bacterial adherence and mucosal inflammation [30, 43, 44]. Therefore, associations of viruses and bacteria could be acting in concert to modify the course of the OME. In the present study, there was an association of detection of HAdV and *S*. *pneumoniae* in adenoid tissues from patients with OME, suggesting that microbial correlations already described in acute middle ear disease [16, 45] could play a role in such chronic processes.

Clinical studies based on microbial detection in patients with adenoid hypertrophy and OME have rarely included a healthy control group. A notable example was a study of biofilms in middle ear samples, including adults and children [25]. Importantly, biofilms were not observed in OME patients, but not in control specimens of middle ear mucosa obtained from patients undergoing cochlear implantation, strongly suggesting that biofilm production is an important factor in development of OME. Through a minimally invasive method (MEW) and a very sensitive procedure (real time PCR), we were able to compare samples of healthy children with those of children with OME. In the light of that result, the findings of some potentially pathogenic bacteria in the middle ear of patients undergoing cochlear implant suggests that their presence in the middle ear is independent of biofilm formation. Unfortunately, in the present study no attempt was made to verify the presence of biofilm.

Concluding, in children with OME and adenoid hypertrophy we observed higher detection rates of potentially pathogenic bacteria, but not respiratory viruses, by real-time PCR in middle ear samples, as compared to control patients without adenoid hypertrophy. We did not observe correlation between the microbiota of adenoid and middle ear, neither in OME children nor in controls. This adds evidence that microbial community present in the middle ear may be associated with persistence of fluid and pathogenesis of OME.

## Supporting information

S1 Table Virus. Primers and probes used for qPCR.
(DOCX)

S2 Table Bacteria. Primers and probes used for qPCR.
(DOCX)
