# Supplementary material for: The pathogens profile in children with otitis media with effusion and adenoid hypertrophy
Source: PLoS One. 2017 Feb 23;12(2):e0171049. doi: 10.1371/journal.pone.0171049 (PMC5322954; doi:10.1371/journal.pone.0171049)
Supplement: S1 Table — Primers and probes used for qPCR. (DOCX) [file pone.0171049.s001.docx]

**S1 Table:** Virus. Primers and probes used for qPCR.

| **Virus or**  **housekeeping gene** | **Primer** | **Sequence 5’-3’** | **Reference** |
| --- | --- | --- | --- |
|  |  |  |  |
|  | A21 | GCTCTTAGCAAAGTCAAGTTGAATGA |  |
| **HRSVA** | A102 | TGCTCCGTTGCATGGTGTATT | [1] |
|  | APB48 | FAM/ACACTCAACAAAGATCAACTTCTGTC/TAMRA |  |
|  |  |  |  |
|  | B17 | GATGGCTCTTAGCAAAGTCAAGTTAA | [1] |
| **HRSV B** | B120 | TGTCAATATTATCTCCTGTACTACGTTGAA |  |
|  | BPB45 | JOE/TGATACATTAAATAAGGATCAGCTGCTGTCATCCA/TAMRA |  |
|  |  |  |  |
|  | Forward | GACCRATCCTGTCACCTCTGAC |  |
| **FLUA** | reverse | AGGGCATTYTGGACAAAKCGTCTA | [2] |
|  | probe | FAM/TGCAGTCCTCGCTCACTGGGCACG/BHQ1 |  |
|  |  |  |  |
|  | Forward | AGATTTGGACCTGCGAGCG |  |
| **RNAse P** | Reverse | GAGCGGCTGTCTCCACAAGT | [2] |
|  | probe | FAM/TTCTGACCTGAAGGCTCTGCGCG/BHQ1 |  |
|  |  |  |  |
|  | INFB-1 | AAATACGGTGGATTAAATAAAAGCAA |  |
| **FLUB** | INFB-2 | CCAGCAATAGCTCCGAAGAAA | [3] |
|  | INFB probe | JOE/CACCCATATTGGGCAATTTCCTATGGC/TAMRA |  |
|  |  |  |  |
|  | Forward | GCACTTCTGTTTCCCC |  |
| **HRV** | reverse | GGCAGCCACGCAGGCT | [4] |
|  | Probe 1 | FAM/AGCCTCATCTGCCAGGTCTA/MGB |  |
|  | Probe 2 | VIC/AGCCTCATCGACCAAACTA/MGB |  |
|  |  |  |  |
|  | HMPVA for | GCCGTTAGCTTCAGTCAATTCAA |  |
|  | HMPVA rev | TCCAGCATTGTCTGAAAATTGC |  |
| **HMPV** | Probe A | FAM/CAACATTTAGAAACCTTCT/MGB | [5] |
|  | HMPVB for | GCTGTCAGCTTCAGTCAATTCAA |  |
|  | HMPVB rev | GTTATCCCTGCATTGTCTGAAAACT |  |
|  | Probe B | FAM/CGCACAACATTTAGGAATCTTCT/MGB |  |
|  |  |  |  |
|  | Forward | GCCACGGTGGGGTTTCTAAACTT |  |
| **HAdV** | Reverse | GCCCCAGTGGTCTTACATGCACATC | [6] |
|  | Probe | FAM/TGCACCAGACCCGGGCTCAGGTACTCCGA/TAMRA |  |
|  |  |  |  |
|  | Forward | GCACAGCCACGTGACGAA |  |
| **HBov** | Reverse | TGGACTCCCTTTTCTTTTGTAGGA | [7] |
|  | Probe | JOE/TGAGCTCAGGGAATATGAAAGACAAGCATCG/TAMRA |  |
|  |  |  |  |
|  | Forward | ACAGATGAAATTTTCAAGTGCTACTTTAGT |  |
| **HPIV1** | Reverse | GCCTCTTTTAATGCCATATTATCATTAGA | [8] |
|  | Probe | FAM/ATGGTAATAAATCGACTCGCT/MGB |  |
|  |  |  |  |
|  | Forward | CTCGAGGTTGTCAGGATATAG |  |
| **HPIV3** | Reverse | CTTGGGAGTTGAACACAGTT | [9] |
|  | Probe | FAM/AATAACTGTAAACTCAGACTTGGTACCTGACTT/TAMRA |  |
|  |  |  |  |
|  | Corona F3 | TGGCGGGTGGGATAATATGT |  |
| **HCov-229E** | Corona R3 | GAGGGCATAGCTCTATCACACTTAGG | [10] |
|  | Corona P2 | VIC/ATAGTCCCATCCCATCAA/MGB |  |
|  |  |  |  |
|  | Corona FOC | CCTTATTAAAGATGTTGACAATCCTGTAC |  |
| **HCov-OC43** | Corona ROC | AATACGTAGTAGGTTTGGCATAGCAC | [10] |
|  | Corona POC | FAM/CACACTTAGGATAGTCCCA/MGB |  |
|  |  |  |  |
|  | Forward | CCCAGCCATGTACGTTGCTA |  |
| **β-actin** | Reverse | TCACCGGAGTCCATCACGAT | [11] |
|  | Probe | VIC/ACGCCTCTGGCCGTACCACTGG/TAMRA |  |
|  |  |  |  |
|  | Forward | GCGGAACCGACTACTTTGGG |  |
| **HEV** | Reverse | CTCAATTGTCACCATAAGCAGCC | [12] |
|  | probe | FAM/TCCGTGTTTCCTTTTATTCTTATAC/MGB |  |

**References**

1. Hu A, Colella M, Tam JS, Rappaport R, Cheng SM. Simultaneous detection, subgrouping, and quantitation of respiratory syncytial virus A and B by real-time PCR. J Clin Microbiol. 2003; 41: 149-154.

2. CDC. CDC. Protocol of real time RT-PCR for influenza A [www.who.int/csr/resources/publications/swineflu/realtimeptpcr/en/index.html2009](http://www.who.int/csr/resources/publications/swineflu/realtimeptpcr/en/index.html2009) [Available from: [www.who.int/crs/resources/publications/swineflu/realtimeptpcr/en/index.html](http://www.who.int/crs/resources/publications/swineflu/realtimeptpcr/en/index.html).

3. van Elden LJ, Nijhuis M, Schipper P, Schuurman R, van Loon AM. Simultaneous detection of influenza viruses A and B using real-time quantitative PCR. J Clin Microbiol. 2001; 39: 196-200.

4. Deffernez C, Wunderli W, Thomas Y, Yerly S, Perrin L, Kaiser L. Amplicon sequencing and improved detection of human rhinovirus in respiratory samples. J Clin Microbiol. 2004; 42: 3212-3218.

5. Kuypers J, Wright N, Corey L, Morrow R. Detection and quantification of human metapneumovirus in pediatric specimens by real-time RT-PCR. J Clin Virol. 2005; 33: 299-305.

6. Heim A, Ebnet C, Harste G, Pring-Akerblom P. Rapid and quantitative detection of human adenovirus DNA by real-time PCR. Journal of medical virology. 2003; 70: 228-239.

7. Neske F, Blessing K, Tollmann F, Schubert J, Rethwilm A, Kreth HW, et al. Real-time PCR for diagnosis of human bocavirus infections and phylogenetic analysis. J Clin Microbiol. 2007; 457: 2116-2122.

8. Kuypers J, Wright N, Ferrenberg J, Huang ML, Cent A, Corey L, et al. Comparison of real-time PCR assays with fluorescent-antibody assays for diagnosis of respiratory virus infections in children. J Clin Microbiol. 2006; 44: 2382-2388.

9. Garbino J, Gerbase MW, Wunderli W, Deffernez C, Thomas Y, Rochat T, et al. Lower respiratory viral illnesses: improved diagnosis by molecular methods and clinical impact. Am J Respir Crit Care Med. 2004; 170: 1197-1203.

10. Kuypers J, Martin ET, Heugel J, Wright N, Morrow R, Englund JA. Clinical disease in children associated with newly described coronavirus subtypes. Pediatrics. 2007; 119: e70-76.

11. Nystrom K, Biller M, Grahn A, Lindh M, Larson G, Olofsson S. Real time PCR for monitoring regulation of host gene expression in herpes simplex virus type 1-infected human diploid cells. J Virol Methods. 2004; 118: 83-94.

12. Proenca-Modena JL, Pereira Valera FC, Jacob MG, Buzatto GP, Saturno TH, Lopes L, et al. High rates of detection of respiratory viruses in tonsillar tissues from children with chronic adenotonsillar disease. PLoS One. 2012;7: e42136.
