# Supplementary material for: The pathogens profile in children with otitis media with effusion and adenoid hypertrophy
Source: PLoS One. 2017 Feb 23;12(2):e0171049. doi: 10.1371/journal.pone.0171049 (PMC5322954; doi:10.1371/journal.pone.0171049)
Supplement: S2 Table — Primers and probes used for qPCR. (DOCX) [file pone.0171049.s002.docx]

**S2 Table:** Bacteria. Primers and probes used for qPCR.

| **BACTERIA** | **PRIMERS** | **PROBES** | **REFERENCES** |
| --- | --- | --- | --- |
| *S. pneumoniae* | 5’TGCAGAGCGTCCTTTGGTCTAT3’ (FORWARD)  5’CTCTTACTCGTGGTTTCCAACTTGA3’ (REVERSE) | FAM 5'TGGCGCCCATAAGCAACACTCGAA-Tamra 3' TAMRA | [1] |
| *S. aureus* | 5'GTTGCTTAGTGTTAACTTTAGTTGTA 3' (FORWARD)  5'AATGTCGCAGGTTCTTTATGTAATTT 3' (REVERSE) | 5'-VIC-AAGTCTAAGTAGCTCAGCAAATGCA- MGB- 3' | [2] |
| *P.aeruginosa* | 5'CGAGTACAACATGGCTCTGG 3' (FORWARD)  5'ACCGGACGCTCTTTACCATA 3' (REVERSE) | 5'-FAM-CCTGCAGCACCAGGTAGCGC-Tamra-3' | [3] |
| *H. influenzae* | 5'CCAGCTGCTAAAGTATTAGTAGAAG 3' (FORWARD)  5'TTCACCGTAAGATACTGTGCC 3' (REVERSE) | 5'-VIC-CAGATGCAGTTGAAGGTTATTTAG -MGB-3' | [4] |
| *M.catarrhalis* | 5'GTCAAACAGCTGGAGGTATTGC 3' (FORWARD)  5'GACATGATGCTCACCTGCTCTA 3' (REVERSE) | 5'-NED- ATCGCAATTGCAACTTT- MGB-3' | [5] |

**References**

1. Corless CE, Guiver M, Borrow R, Edwards-Jones V, Fox AJ, Kaczmarski EB. Simultaneous detection of Neisseria meningitidis, Haemophilus influenzae, and Streptococcus pneumoniae in suspected cases of meningitis and septicemia using real-time PCR. J Clin Microbiol. 2001; 39: 1553-1558.

2. Kilic A, Basustaoglu AC. Double triplex real-time PCR assay for simultaneous detection of Staphylococcus aureus, Staphylococcus epidermidis, Staphylococcus hominis, and Staphylococcus haemolyticus and determination of their methicillin resistance directly from positive blood culture bottles. Res Microbiol. 2011; 162: 1060-1066.

3. Feizabadi MM, Majnooni A, Nomanpour B, Fatolahzadeh B, Raji N, Delfani S, et al. Direct detection of Pseudomonas aeruginosa from patients with healthcare associated pneumonia by real time PCR. Infect Genet Evol. 2010; 10: 1247-1251.

4. Abdeldaim GM, Stralin K, Kirsebom LA, Olcen P, Blomberg J, Herrmann B. Detection of Haemophilus influenzae in respiratory secretions from pneumonia patients by quantitative real-time polymerase chain reaction. Diagn Microbiol Infect Dis. 2009; 64: 366-373.

5. Heiniger N, Spaniol V, Troller R, Vischer M, Aebi C. A reservoir of Moraxella catarrhalis in human pharyngeal lymphoid tissue. J Infect Dis. 2007; 196: 1080-1087.
